# Results of Induction of Labor with Prostaglandins E1 and E2 (The RIPE Study): A Real-World Data Analysis of Obstetrical Effectiveness and Clinical Outcomes of Pharmacological Induction of Labor with Vaginal Inserts

**DOI:** 10.3390/ph16070982

**Published:** 2023-07-08

**Authors:** Maciej W. Socha, Wojciech Flis, Miłosz Pietrus, Mateusz Wartęga

**Affiliations:** 1 Department of Perinatology, Gynecology and Gynecologic Oncology, Faculty of Health Sciences, Collegium Medicum in Bydgoszcz, Nicolaus Copernicus University, Łukasiewicza 1, 85-821 Bydgoszcz, Poland; 2 Department of Obstetrics and Gynecology, St. Adalbert’s Hospital in Gdańsk, Copernicus Healthcare Entity, Jana Pawła II 50, 80-462 Gdańsk, Poland; 3 Department of Gynecology and Oncology, Jagiellonian University Medical College, 31-501 Kraków, Poland; 4 Department of Pathophysiology, Faculty of Pharmacy, Collegium Medicum in Bydgoszcz, Nicolaus Copernicus University, M. Curie- Skłodowskiej 9, 85-094 Bydgoszcz, Poland; mwartega@cm.umk.pl

**Keywords:** induction of labor, patients’ clinical outcomes, real-world data, obstetrics, labor, delivery, pregnancy outcome, Misoprostol, Dinoprostone

## Abstract

Despite extensive knowledge of the mechanisms responsible for childbirth, the course of labor induction is often unpredictable. Therefore, labor induction protocols using prostaglandin analogs have been developed and tested to assess their effectiveness in labor induction unequivocally. A total of 402 women were collected into two groups—receiving vaginal Misoprostol or vaginal Dinoprostone for induction of labor (IOL). Then, the patients were compared in groups depending on the agent they received and their gestational age. Most patients delivered within 48 h, and most of these patients had vaginal parturition. Patients who received the Dinoprostone vaginal insert required statistically significantly more oxytocin administration than patients who received the Misoprostol vaginal insert. Patients who received the Misoprostol vaginal insert used anesthesia during labor statistically more often. Patients who received Misoprostol vaginal inserts had a statistically significantly shorter time to delivery than those with Dinoprostone vaginal inserts. The prevalence of hyperstimulation was similar in all groups and remained low. Vaginal Misoprostol-based IOL is characterized by a shortened time to delivery irrespective of the parturition type, and a lower need for oxytocin augmentation, but also by an increased demand for intrapartum analgesia administration. A vaginal Dinoprostone-based IOL protocol might be considered a more harmonious and desirable option in modern perinatal care.

## 1. Introduction

Induction of labor is defined as an attempt to induce regular uterine contractions that will result in vaginal delivery within 24–48 h [1]. This procedure is currently the most frequently performed operation in modern obstetrics—over 20% of pregnant women undergo IOL [2]. Furthermore, it is postulated that the percentage of labor induction will increase in current obstetrical practice [3]. In current obstetrics worldwide, the gold standard IOL protocol consists of intravenous oxytocin infusion. Additionally, due to an unfavorable cervix, most women require prior cervical preparation with a cervical ripening agent. Therefore, the typical IOL protocol most commonly involves the use of a cervical ripening agent followed by intravenous oxytocin infusion as the next step. Mechanical devices, progesterone antagonists, and prostaglandins are widely used to ripen the unprepared cervix with different efficacy and safety profile rates [4,5,6,7].

Considering prostaglandins (PGs) for IOL, the most commonly used substances are Misoprostol (prostaglandin E1 analog) and Dinoprostone (prostaglandin E2 analog) [8]. Despite their similar action profiles, there are many pharmacokinetic differences between them. Their effects are dose- and route-dependent, including cervical ripening and dilation [9]. Misoprostol can induce systolic uterine activity, while Dinoprostone has only trace effects on uterine contractions. Although Misoprostol is not licensed for labor induction, it is widely used successfully off-label for IOL worldwide [3]. While Dinoprostone is available in two formulations (vaginal insert or vaginal gel), Misoprostol can be administered via several possible routes (oral, rectal, sublingual, and vaginal) [7,8,9,10]. Misoprostol and Dinoprostone are highly effective cervical ripening agents in women with an unfavorable cervix [10,11]. However, it has not yet been established which prostaglandin analogs are more effective in IOL and have a lower rate of side effects. The main side effect of using PGs is uterine tachysystole accompanied by cardiotocographic abnormalities with nonreassuring fetal heart rate patterns [12].

This study aims to analyze the effectiveness of intravaginal inserts containing the PG analogs Misoprostol and Dinoprostone in IOL, with additional emphasis on perinatal care, i.e., possible side effects and pain management. In this study, we analyze retrospective data from actual patients from one of the biggest perinatology centers in this part of Poland, which reflects the results of real-life obstetrical practice. An inherent feature of every induction of labor is cervical ripening, which is why the study is called The RIPE Study, referring to the matured cervix. This acronym incorporates the title’s first letters and evokes thoughts of a ripened cervix. This dual meaning not only makes the title memorable but also accurately reflects the focus and objectives of the study.

## 2. Results

A total of 402 pregnant women (34 to 42 weeks of gestation) who had induction of labor were included in the study. Subsequently, the 402 initially selected patients were divided into two groups according to the prostaglandin analog (Dinoprostone or Misoprostol) used for induction of labor. A total of 190 patients in the study received a vaginal insert with Misoprostol, and the remaining 212 pregnant women received an insert with Dinoprostone. The patients were in full-term pregnancy (>37 weeks of gestation)—n = 162, preterm pregnancy (below 37 weeks of gestation)—n = 95, and post-term pregnancy (>41 weeks of gestation)—n = 145. All patients were adults.

**Figure 1:**
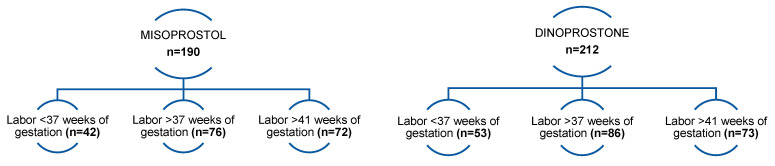
Patients finally enrolled for the study were divided into groups depending on the vaginal insert used and divided into subgroups depending on their gestational age.

In addition, in each of the two groups, the following subgroups were taken into account: patients who gave birth after up to 37 weeks of gestation (preterm delivery)—n = 42 and n = 53, patients who gave birth after between 37 and 41 weeks of gestation—n = 76 and n = 86, and those who gave birth after 41 weeks of pregnancy—n = 72 and n = 73 (Figure 1). Then, analysis was performed in individual subgroups. The patients ranged from 18 to 39 years old, with a median age of 27 years. In addition, 244 patients (62%) were primiparas, and the remaining 158 (38%) were multiparous patients.

The mean gestational age in the Dinoprostone group, depending on the gestational age, was 35.2 in the group of preterm labor patients (ranged from 34.0 to 36.0), 38.6 (ranged from 37.0 to 40.0) in the group of patients who delivered after between 37 and 41 weeks of gestation, and 41.2 (ranged from 41.0 to 42.0) in the group of overdue pregnancy patients. The corresponding values in the Misoprostol group were 35.1 (ranged from 34.0 to 36.0), 38.5 (ranged from 37.0 to 40.0), and 41.1 (ranged from 41.0 to 42.0), respectively. No statistically significant differences were found.

There were (Table 1) 34 (64.2%) vaginal deliveries (VDs) in the Dinoprostone group in the preterm delivery group, 63 (73.3%) in the group with term delivery and 46 (63.0%) in the group with post-term delivery. The corresponding values in the Misoprostol group were 54.8% vs. 57.9% vs. 61.1%, respectively. Surprisingly, in the Dinoprostone vaginal insert group, there were statistically significantly more VDs in the full-term subgroup (37 to 41 weeks of gestation)—*p* = 0.0394.

In the Dinoprostone group, of the total of 53 patients who had preterm delivery, 81.1% (n = 43) required oxytocin augmentation (Table 2). In the Misoprostol preterm delivery group, 35.7% (n = 15) required oxytocin augmentation. The necessity for oxytocin augmentation was statistically significantly more frequent in the Dinoprostone group in terms of labor before 37 weeks of gestation (*p* < 0.0001). In the Dinoprostone term delivery group (37 to 41 weeks of pregnancy), 66.3% (n = 57) required oxytocin administration, while in the Misoprostol term delivery group, only 19.7% (n = 15) required it. The requirement of oxytocin administration was statistically significantly more frequent in the Dinoprostone term delivery group compared with the Misoprostol term delivery group (*p* < 0.0001). Of the total of 73 patients in the post-term delivery group who had a Dinoprostone vaginal insert, 58.9% (n = 43) required oxytocin augmentation. In the Misoprostol group, only 11.1% (n = 8) required oxytocin augmentation. Oxytocin administration was statistically significantly more frequent in the Dinoprostone group compared with the Misoprostol group (*p* < 0.0001).

Considering the need for intravenous analgesia in preterm labor, 75.5% (n = 40) of the patients in the Dinoprostone group required Remifentanil, and in the Misoprostol group 92.9% (n = 39) required it. Intravenous analgesia was statistically significantly more frequent in the preterm labor group in which Misoprostol was used compared with the Dinoprostone group (*p* = 0.0245).

Of the 86 in the Dinoprostone term delivery group, 70.9% (n = 61) required intrapartum analgesia, and in the Misoprostol group, 92.1% (n = 70) required it. The need for intrapartum analgesia was statistically significantly higher in the Misoprostol group compared with the Dinoprostone group (*p* = 0.0006).

In the Dinoprostone post-term delivery group (>41 weeks of gestation), 68.5% (n = 50) required intravenous analgesia. In the Misoprostol group, 88.9% (n = 64) required it. The need for intrapartum analgesia was statistically significantly higher in the Misoprostol group compared with the Dinoprostone group (*p* = 0.0027).

The percentage of tachysystole remained at approximately similar levels in each group. There were no significant differences between the groups in the incidence of tachysystole (*p* > 0.05). A comparative analysis is visualized in Table 2.

Here we would like to present an analysis of the data in each individual gestational age group.

### 2.1. Before 37 Weeks (Table 3; Table 4; Table 5)

Of the 53 patients who delivered prematurely (VD and CS) and received the Dinoprostone insert, 17.0% (n = 9) delivered within 24 h compared with the Misoprostol group, for which this value was 64.3% (n = 27) (Table 3). A total of 60.4% (n = 32) had delivery in 24–48 h in the Dinoprostone group, compared with the Misoprostol group, for which this value was 14.3% (n = 6). Finally, 22.6% (n = 12) had a time to delivery >48 h in the Dinoprostone group, as compared with the Misoprostol group, for which this value was 21.4% (n = 9). The time to delivery was statistically significantly shorter in the Misoprostol group (*p* < 0.0001).

There were 34 vaginal deliveries (64.2%) in the Dinoprostone group, compared with 23 (54.8%) in the Misoprostol group. The respective cesarean section rates were 35.8% vs. 45.2%. However, no statistically significant differences for those variables were found (*p* = 0.3536).

Of the total of 34 VDs in the Dinoprostone group, 14.7% (n = 5) delivered within 24 h, compared with the Misoprostol group, for which the corresponding value was 65.2% (n = 15) (Table 4). In the Dinoprostone group, 64.7% (n = 22) had vaginal delivery in 24–48 h, compared with 21.7% (n = 5) in the Misoprostol group. Only 20.6% (n = 7) had a time to VD >48 h in the Dinoprostone group, while in the Misoprostol group, 13.0% (n = 3) did. The time to VD labor was statistically significantly shorter in the Misoprostol group (*p* = 0.0004).

Of the 19 cesarean deliveries in the Dinoprostone group, 21.1% (n = 4) delivered within 24 h, as compared with the Misoprostol group, for which this value was 63.2% (n = 12) (Table 5). A total of 52.6% (n = 10) in the Dinoprostone group had time to CS delivery of 24–48 h, compared with the Misoprostol group, for which this value was 5.3% (n = 1). Finally, 26.3% (n = 5) delivered after 48 h in the Dinoprostone group, compared with the Misoprostol group, for which this value was 31.6% (n = 6). The time to cesarean delivery was statistically significantly shorter in the Misoprostol group (*p* = 0.0033).

### 2.2. Between 37 and 41 Weeks of Gestation

There were 63 vaginal deliveries (73.3%) in the Dinoprostone group, compared with 44 (57.9%) in the Misoprostol group (Table 6). The respective cesarean section rates were 26.7% vs. 42.1%. The Dinoprostone group had statistically significantly more VDs than the Misoprostol group regarding term labor (*p* = 0.0394).

Of the total of 86 term deliveries (both vaginal and cesarean) in the Dinoprostone group, 33.7% (n = 29) delivered within 24 h, as compared with the Misoprostol group, for which this value was 80.3% (n = 61). A total of 53.5% (n = 46) had a time to delivery of 24–48 h in the Dinoprostone group, as compared with the Misoprostol group, for which this value was 9.2% (n = 7). Only 12.8% (n = 11) had time to delivery >48 h in the Dinoprostone group, as compared with the Misoprostol group, for which this value was 10.5% (n = 8).

The time to delivery was statistically significantly shorter in the Misoprostol group (*p* < 0.0001).

Of the total of 63 vaginal term deliveries in the Dinoprostone group, 31.7% (n = 20) of patients delivered vaginally within 24 h, as compared with the Misoprostol group, for which this value was 93.2% (n = 41) (Table 7). A total of 58.7% (n = 37) had a time to VD of 24–48 h in the Dinoprostone group, while in the Misoprostol group, the corresponding value was 2.3% (n = 1). Only 9.5% (n = 6) had time to VD >48 h in the Dinoprostone group, as compared with the Misoprostol group, for which this value was 4.5% (n = 2). The time to VD was statistically significantly shorter in the Misoprostol term delivery group compared with the Dinoprostone group (*p* < 0.0001).

Of the total of 23 CSs in the Dinoprostone term delivery group, 39.1% (n = 9) delivered in 24 h, compared with the Misoprostol group, for which this value was 62.5% (n = 20) (Table 8). Considering the time to CS of within 24–48 h, 39.1% (n = 9) delivered in the Dinoprostone group, as compared with the Misoprostol group, for which this value was 18.8% (n = 6). A total of 21.7% (n = 5) delivered after 48 h in the Dinoprostone group, as compared with the Misoprostol group, for which this value was 18.8% (n = 6). No statistically significant differences were found for those variables (*p* = 0.1752).

### 2.3. >41 Weeks of Gestation

There were 46 VDs (63.0%) in the Dinoprostone post-term delivery group, compared with 44 (61.1%) in the Misoprostol group (Table 9). The CS rates were 37.0% and 38.0%, respectively. No statistically significant differences were found (*p* = 0.8134).

Of the total of 73 deliveries (both CS and VD) in the Dinoprostone group, 41.1% (n = 30) delivered within 24 h, as compared with the Misoprostol group, for which this value was 88.9% (n = 64). A total of 45.2% (n = 33) delivered within 24–48 h in the Dinoprostone group, compared with the Misoprostol group, for which this value was 6.9% (n = 5). A total of 13.7% (n = 10) delivered after 48 h in the Dinoprostone group, as compared with the Misoprostol group, for which this value was 4.2% (n = 3). The time to delivery was statistically significantly shorter in the Misoprostol post-term delivery group (*p* < 0.0001).

Of the total of 46 post-term VDs in the Dinoprostone group, 41.3% (n = 19) had VD within 24 h, compared with the Misoprostol group, for which this value was 97.7% (n = 43) (Table 10). A total of 56.5% (n = 26) of patients delivered vaginally in 24–48 h in the Dinoprostone group, compared with the Misoprostol group, for which this value was 2.3% (n = 1). Surprisingly, 2.2% (n = 1) delivered after 48 h in the Dinoprostone group, compared with the Misoprostol group, for which this value was 0.0% (n = 0). The time to VD was statistically significantly shorter in the Misoprostol group (*p* < 0.0001) compared with the Dinoprostone group.

Of the 27 cesarean deliveries in the post-term Dinoprostone group, 40.7% (n = 11) delivered in up to 24 h, compared with the Misoprostol group, for which this value was 75.0% (n = 21) (Table 11). A total of 25.9% (n = 7) of patients delivered in 24–48 h in the Dinoprostone group, compared with the Misoprostol group, for which this value was 14.3% (n = 4). Only 33.3% (n = 9) delivered after >48 h in the Dinoprostone group, in comparison with the Misoprostol group, for which this value was 10.7% (n = 3). The time to cesarean delivery was statistically significantly shorter in the case of the post-term Misoprostol cesarean deliveries (*p* = 0.0313).

## 3. Discussion

One in five women (approximately 20%) undergo labor induction for both maternal and fetal indications. Oxytocin, the gold standard in labor induction, has an insignificant effect on cervical ripening despite its great effectiveness in triggering uterine contractions. Therefore, proper cervical preparation is the key to a correct and successful IOL. Prostaglandin analogs appear to be excellent pharmacological agents for labor induction due to their significant effect on cervical ripening and their ability to trigger uterine contractions [8,13,14,15]. However, it has not been possible to determine which prostaglandin analogs dominate in effectiveness and efficiency. Data from various centers and publications often show conflicting results and clinical practice does not always follow the data from the professional literature. The rate of labor induction has been steadily increasing over the last few decades, with an estimated further increase in the coming years [16]. Additional burdens and severe pregnancy complications pose new challenges for medical staff, which they must efficiently meet. A crucial issue is providing adequate perinatal care during a pregnant patient’s stay and childbirth. In addition, the implementation of high-level nursing care greatly contributes to a quick return to work, which has an additional positive effect on psychological comfort and does not eliminate the patient from social life [17,18,19].

This study aimed to evaluate the efficacy of Misoprostol and Dinoprostone vaginal inserts in achieving vaginal delivery. Efficacy was demonstrated for individual groups because regardless of the IOL agent used, the vast majority of women delivered within 48 h, and most of these women had vaginal parturition (64.2%, 73.3%, and 63% for the Dinoprostone vaginal insert vs. 54.8%, 57.9%, and 61.1% for the Misoprostol vaginal insert). It is also worth noting that statistical significance was demonstrated for a greater number of vaginal deliveries in the group of patients who gave birth at term after using the Dinoprostone insert. These data are consistent with previously published studies [20,21,22].

What is extremely interesting is that our study showed that, regardless of gestational age, patients who received the Dinoprostone insert required statistically more oxytocin administration than patients who received the Misoprostol vaginal insert (81.1%, 66.3%, and 58.9% vs. 35.7%, 19.7%, and 11.1%, respectively). In addition, it is worth noting that even in the case of the need for oxytocin stimulation in the group of patients who received Misoprostol, the percentage of patients requiring stimulation remained very low. Misoprostol is a more potent agent and more often leads to the initiation of contractions resulting in labor. This labor usually started, and the patients delivered, within 24 h after insertion of the Misoprostol vaginal insert. In turn, patients with Dinoprostone required additional oxytocin, which allowed them to achieve delivery. The definition of labor induction, i.e., achieving labor within 48 h, was conducted with similar success in both groups. However, in the Misoprostol group, the addition of oxytocin was mainly unnecessary, and delivery occurred dynamically before 24 h. In the Dinoprostone group, oxytocin was added, and delivery took place harmoniously, mainly after 24 h but still within 48 h. The most significant demand for oxytocin occurred in the case of preterm labor (regardless of the vaginal insert administered)—81.1% and 35.7%, respectively. The above data may suggest that in preterm labor, both Misoprostol and Dinoprostone have reduced potential to generate uterine contractions. We believe that a possible explanation for this phenomenon lies in the histology and molecular biology of the uterine muscle. Prostaglandins act via membrane prostaglandin receptor isoforms (EP 1 to EP 4) [23,24]. During pregnancy, the concentration of prostaglandin receptors remains low, with a peak increase at term [9,24]. Therefore, we suggest that during the course of preterm labor (<37 weeks of gestation), the expression of prostaglandin receptors may not be sufficient to ensure adequate generation of contractile activity by prostaglandin analogs. We believe it is worth conducting additional studies to evaluate the effect of prostaglandin analogs in the induction of preterm labor.

In each of the study groups, the majority of patients showed a willingness to use intrapartum analgesia (intravenous Remifentanil). However, regardless of the gestational age, patients who received the Misoprostol vaginal insert statistically more often used anesthesia during labor (92.9%, 92.1%, and 88.9% vs. 75.5%, 70.9%, and 68.5%). These data are consistent with previous studies. According to these studies, patients receiving a vaginal insert with Misoprostol more often required intrapartum analgesia with opioids [25,26,27]. However, due to the study design, these figures may be overestimated. The differences in the results may also be due to the fact that no cut-off threshold for the use of analgesia was adopted in our study. In addition, the multidimensional nature of the pain experience renders pain assessment challenging. Therefore, objectively assessing the actual severity of pain is highly problematic. It is also worth noting that the patients received anesthesia based on their subjective pain evaluation. We suggest that pregnant patients (especially in the Polish population) have insufficient information about nonpharmacological pain relief methods. Therefore, patients immediately reach for pharmacological approaches (intravenous or epidural) in pain, omitting nonpharmacological, highly effective ways [28,29,30,31,32,33]. Increasing patients’ awareness about possible nonpharmacological analgesia methods during childbirth is crucial. Midwives are vital in providing obstetric services to women globally [34]. Besides pharmacological and nonpharmacological treatment, midwives can also give support through praise, therapeutic touch, and explaining or informing women of labor progress [35,36]. However, inadequate resources and stressful work settings cause midwife burnout and hinder adequate labor pain management [37,38]. Therefore, it is crucial to maintain highly educated and devoted obstetrical staff to improve labor pain management.

In our study protocol, patients received an intravenous infusion of Remifentanil. It should also be remembered that IV anesthesia (Remifentanil) used during labor is off-label. The gold standard in obstetrics worldwide is epidural anesthesia. Recent studies revealed that, although intravenous anesthesia provides satisfactory pain relief, epidural analgesia provides better analgesia and satisfaction than analgesia with Remifentanil [39,40]. This type of anesthesia was used due to epidural anesthesia’s unavailability (during the study period) due to procedural factors. Considering the above, we plan to conduct additional studies on using vaginal inserts with prostaglandin analogs and epidural anesthesia to evaluate their effectiveness. Since, in many hospitals in Poland, the availability of epidural anesthesia is limited, we wanted to share actual data from our practice using Remifentanil.

Considering drug safety, hyperstimulation (tachysystole) occurred in each group with a similar frequency and remained below 10%, regardless of the IOL agent used. The rate of tachysystole was not statistically significant. The presented data indicate that in our practice, both Dinoprostone and Misoprostol had a low rate of uterine hyperstimulation. This is in contrast to published studies that suggest that prostaglandin analogs (especially Misoprostol) may be associated with increased rates of hyperstimulation [26,41,42,43]. We assume an explanation may be the practical fact that physicians paid more attention to nonreassuring fetal heart rate patterns when diagnosing them as an indication for emergency cesarean section for threatened asphyxia rather than hyperstimulation. We believe that this exciting topic requires further research.

Considering the group of patients who gave birth prematurely (<37 weeks of gestation), statistical significance was demonstrated for shortening the time to delivery (both vaginal and cesarean section) for patients who received intravaginal Misoprostol (*p* < 0.0001 and *p* = 0.0004, respectively). At the same time, no differences were found for the delivery route depending on the vaginal agent used. In addition, most patients who received Misoprostol delivered within 24 h (64.3%), while most patients who received Dinoprostone gave birth within 24–48 h (60.4%).

In the group of patients who delivered at term (37–41 weeks of gestation), the statistical significance of shortening the time to delivery was demonstrated in patients who received intravaginal Misoprostol (*p* < 0.0001) regardless of the mode of delivery. Again, the vast majority of patients who received Misoprostol delivered within 24 h (80.3%), while the majority of patients who received Dinoprostone delivered within 24–48 h (53.5%).

In the group of patients who delivered after term (>41 weeks of gestation), the statistical significance of shortening the time to delivery (both vaginal and cesarean) was demonstrated in patients who received intravaginal Misoprostol (*p* < 0.0001). At the same time, no differences were found for the delivery route depending on the vaginal agent used. In addition, most patients who received Misoprostol delivered within 24 h (88.9%), while most patients who received Dinoprostone delivered within 24–48 h (45.2%).

No statistical differences were found for the delivery route depending on the vaginal agent used, except for the group of patients at term who received Dinoprostone. This group was characterized by a statistically significantly higher percentage of vaginal deliveries (*p* < 0.0394).

The presented data are consistent with the studies conducted so far on the effectiveness of the use of prostaglandins in labor induction [44,45,46].

Summing up all the above data, a noticeable pattern emerges. Using both Misoprostol and Dinoprostone (in the form of vaginal inserts) effectively achieves labor within 48 h. This is consistent with the accepted definition of successful labor induction [1,47,48]. However, the use of Misoprostol (regardless of the gestational age) is characterized by a shortened time to delivery irrespective of the parturition type (most patients delivered within 24 h). Comparing both protocols, Misoprostol-based protocols were associated with less oxytocin augmentation but increased demand for intrapartum opioid analgesia on demand. A possible explanation for the increased need for anesthesia in this group may be that these patients deliver in a shorter period (up to 24 h) compared with those using Dinoprostone, which makes labor more violent and tumultuous, which in turn causes an increased need for anesthesia. On the other hand, the use of Dinoprostone (regardless of the gestational age) resulted in most patients giving birth within 24–48 h. In addition, most of the patients treated with Dinoprostone-based protocols had oxytocin augmentation. Finally, using Dinoprostone was associated with a statistically lower demand for intrapartum anesthesia. Considering both labor induction regimens—vaginal Dinoprostone vs. vaginal Misoprostol—the use of a Misoprostol protocol is characterized by a significant shortening of the time to delivery. However, considering the defining criterion of labor induction, a more desirable management model for patients seems to be a regimen with Dinoprostone. Using a regimen with Dinoprostone leads to results similar to those of Misoprostol of delivery within 48 h, but with a lower need for anesthesia.

It is also worth noting that the route of administration of a given agent for labor induction may have a significant impact on the course of labor. Our study evaluated only Misoprostol and Dinoprostone vaginal inserts. Some studies additionally compare the effectiveness of using Misoprostol orally or sublingually. Referring to these studies, the application of oral or sublingual Misoprostol, with similar efficacy to vaginal Misoprostol, seems to be associated with greater patient satisfaction. We believe that greater patient satisfaction can translate into a less violent course of delivery despite the use of Misoprostol [49,50,51]. We believe this is an excellent starting point for further in-depth studies that will compare the effectiveness of both Misoprostol in various forms and Dinoprostone vaginal inserts.

The use of Dinoprostone in the group of full-term pregnant patients was characterized by a higher percentage of vaginal deliveries. We believe that the use of Dinoprostone (despite a slight extension of the time to delivery) determines a more peaceful and harmonious course of childbirth, which undoubtedly translates into the patient’s well-being and satisfaction. Moreover, such a protocol is associated with an increase in the percentage of vaginal deliveries, especially in full-term pregnancies. A more harmonious birth still falls within the definition of successful labor induction but also leads to less need for intrapartum anesthesia, less trauma, and a more positive birthing experience. In addition, we believe that a more harmonious birth leads to faster postpartum recovery, significantly affecting patients’ quality of life.

We believe that the differences between Dinoprostone and Misoprostol shown in our study result strictly from their pharmacodynamics. The entire ripening process is complex, consisting of an inflammatory reaction and enzymatic degradation [52].

Prostaglandins act through prostaglandin receptors—EP1, EP2, EP3, and EP4—which are a G protein-coupled cell membrane receptor family.

EP2 and EP4 receptors are coupled with Gs protein, stimulating (via increased cAMP production) muscle relaxation. On the other hand, EP1 and EP3 receptors are responsible for muscle contraction. The EP1 receptor is associated with the Gq protein, while the EP3 receptor is associated with the Gi protein—its stimulation causes a decrease in cAMP concentration. During the perinatal period, the expression of COX-2 and prostaglandin receptors is enhanced in the extracellular matrix of the cervix [53,54]. These receptors (along with COX-2) are also expressed in fetal membranes, myometrial cells, cervical tissue, and decidua [55]. Considering the above, we believe that the increase in the expression of prostaglandin receptors in the myometrial cells and decidua in the perinatal period may be significantly related to the triggering of contractile activity after the application of a vaginal insert with prostaglandin analogs.

In addition, it has been shown that the receptor with the highest concentration in the cervical tissue at term is the EP4 receptor [56,57]. Evidence shows that it is the activation of this receptor that is responsible for the regulation of cervical ripening by prostaglandins. Our study showed that both analogs are effective; however, after using Dinoprostone, more patients delivered within 24–48 h, and in this group, there was a higher rate of vaginal deliveries. Additionally, patients who received a Dinoprostone insert required significantly more oxytocin augmentation than Misoprostol receivers. These differences may be due to the distribution of prostaglandin receptors and the prostaglandin analog itself. Studies have shown that the dominant prostaglandin in the cervical ripening process is prostaglandin PGE2 [24]. In addition, the primary receptor involved in cervical ripening is the EP4 receptor [57]. Dinoprostone (a PGE2 analog) shows the highest affinity to the EP2 and EP4 receptors and slightly lower affinity to other prostaglandin receptors [58].

In comparison, Misoprostol shows great affinity for EP1 and EP3 receptors [8,12,58]. From the above data, it can be concluded that Dinoprostone, being an analog of PGE2 (the main prostaglandin responsible for cervical ripening) with the highest affinity for the receptors that stimulate the relaxation of muscle cells and actively participate in the ripening of the cervix (EP4 receptor), has a much more significant effect on the cervix than Misoprostol (an analog of PGE1). Due to the low affinity for the EP1 and EP3 receptors and high affinity for the EP2 and EP4 receptors, the use of Dinoprostone resulted in slightly longer labor and a greater need for oxytocin augmentation. Therefore, we believe that the use of Dinoprostone led to a much better preparation of the cervix, which translated into more gentle and harmonious deliveries, most of which were vaginal deliveries. On the other hand, using Misoprostol (which has a high affinity for the EP1 and EP3 receptors) resulted in a shorter time to labor and a lower need for oxytocin augmentation. A possible explanation for this phenomenon may be Misoprostol’s influence on specific receptors, which contributed to a greater extent to triggering stronger contractions (due to which patients required oxytocin stimulation in a smaller percentage), translating into a shorter delivery time.

To the best of our knowledge, this is the first study to show an accurate timing relationship between the two agents while highlighting the potential benefit of a slightly longer time to delivery when using a labor induction protocol with a Dinoprostone vaginal insert. Other studies in this field promote using Misoprostol as an agent that allows one to achieve labor in a shorter time [59]. We believe our study may help clinicians select a more appropriate agent to induce labor based on their needs. Previous studies comparing prostaglandin analogs contradict our results, showing that both prostaglandin analogs have similar efficacy and cesarean section rates [59,60]. However, these studies do not consider the exact grouping by week of gestation. Therefore, we believe that our study can be an excellent starting point for further consideration in this area.

Our study has its limitations, such as the lack of division of patients into primiparas and multiparous women and the lack of division into indications for cesarean delivery. Additionally, due to the retrospective nature of our study, the results should be interpreted cautiously, as there is no control group. Furthermore, birth safety in terms of neonatal outcomes was not assessed. We believe that it is worth conducting further studies that, apart from the effectiveness of prostaglandin analogs in labor induction, also assess the birth status of newborns. Other labor induction methods and the assessment of additional clinical parameters are subject to continuous analysis by the research team.

## 4. Materials and Methods

Medical staff recorded data on labor induction in a computer database of the hospital-administered system during the patient’s stay. These data were collected and stored in the hospital computer system, where they were archived, which ensured their cybersecurity. Then, based on the stored data, analysis was performed. During the analysis of the previously obtained documentation, the following information was obtained: duration of pregnancy (determined based on the date of the last menstruation, confirmed using first-trimester ultrasonography), type of cervical ripening agent used, course of the induction of labor, duration of labor, route of delivery, previous obstetric history, and patient demographic data. In addition, the previously acquired dataset was checked for inconsistencies and possible errors. The criteria for inclusion in the study group were the following: the use of Misoprostol or Dinoprostone vaginal inserts as induction of labor agents, delivery between 34 and 42 weeks of gestation, single live pregnancy, cephalic fetal presentation, and no previous cesarean sections. The study included patients qualified for induction of labor for maternal and fetal indications and with an unfavorable cervix (Bishop score <6 and >2). In addition, clinical indications for IOL followed the current recommendations of the Polish Society of Gynecologists and Obstetricians [61].

**Figure 2:**
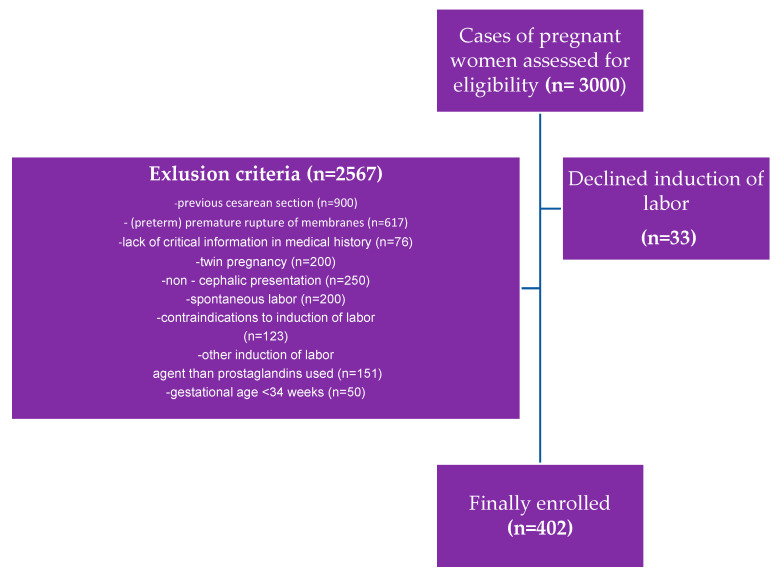
Inclusion and exclusion criteria for the study population.

The exclusion criteria were the following: the onset of spontaneous labor, previous cesarean section, premature rupture of membranes, preterm premature rupture of membranes, twin pregnancy, lack of critical information in medical documentation, and any contraindications to vaginal delivery and induction of labor following the Polish guidelines. An analysis of the documentation covering 3002 deliveries at the analyzed time was carried out, of which, based on the adopted criteria, 402 cases were qualified for study (Figure 2).

Induction of labor protocol consisted of 200 micrograms of Misoprostol or 10 milligrams of Dinoprostone in the form of vaginal inserts in all included patients. In the absence of labor, defined as regular contractions within 24 h of insertion of vaginal insert, oxytocin augmentation was used in the local low-dose protocol. Tachysystole was diagnosed based on the following criteria: contraction lasting >2 min, no significant interval between contractions, and >5 contractions in 10 min. In the event of hyperstimulation (tachysystole), an intravenous infusion of 25 micrograms of Fenoterol was used, and the previously administered vaginal insert was removed. During labor, the patients were given intrapartum analgesia as an intravenous infusion of Remifentanil on demand.

We assessed the effectiveness of triggering uterine contraction activity after administering a vaginal insert with Misoprostol and Dinoprostone. The primary outcome was to evaluate the time from vaginal insert (with Dinoprostone or Misoprostol) administration to delivery (both vaginal and cesarean) within 24–48 h. The secondary outcomes were the proportion of women undergoing cesarean section, oxytocin augmentation, and the necessity for intrapartum analgesia application. In addition, we compared the groups regarding the percentage of vaginal births and the rate of cesarean sections. Finally, we also compared the groups regarding the occurrence of uterine hyperstimulation (tachysystole) and the need for intravenous intrapartum analgesia.

### 4.1. Ethical Review

In accordance with the decision of the Institutional Ethics Committee of Collegium Medicum in Bydgoszcz, Nicolaus Copernicus University (KB 303/2020 and annexes), retrospective anonymized medical data were analyzed. In this case, the patients’ informed consent to participate in the retrospective data analysis was not required. Regardless, each patient consented to the induction of labor procedure under local law.

### 4.2. Statistical Analysis

The statistical analysis was performed using Microsoft Excel software and the statistical suite StatSoft. Inc. (2014). STATISTICA (data analysis software system)—version 12.0 was used.

The quantitative variables were characterized by the arithmetic mean of standard deviation or median or max/min (range) and 95% confidence interval. The qualitative variables were presented with the use of count and percentage.

The W Shapiro–Wilk test was used to check if a quantitative variable derived from a normal distribution population. To prove the hypotheses on the homogeneity of variances, Leven (Brown–Forsythe) test was utilized. The statistical significance of differences between the two groups was processed with the t-Student test or U Mann–Whitney test. The significance of the difference between more than two groups was assessed with the F test (ANOVA) or Kruskal–Wallis. Regarding statistically significant differences between the two groups, post hoc tests were utilized (Tukey test for F or Dunn for Kruskal–Wallis). Model t-Student or Wilcoxon signed-rank tests were used for two paired variables. The significance of the difference between more than two variables in the paired variables model was checked using analysis of variance with repeated measurements or using the Friedman test. Chi-squared tests for independence were used for qualitative variables. Correlation analysis was used to determine dependence, strength, and direction between variables by determining Pearson’s or Spearman’s correlation coefficients. The statistical significance level of *p* = 0.05 was used in all the calculations.

## 5. Conclusions

The RIPE Study enhances our understanding of the effectiveness of vaginal inserts containing PG analogs: Misoprostol and Dinoprostone. Our real-world data are based on the results of actual patients and reflect the true meaning of pharmacological agents used in obstetrical practice. Considering the pharmacodynamics of Misoprostol, with its strong uterotonic effect, leading to dynamic labor progress, it can be assumed that the contractions were so strong that patients more often required analgesics. Another essential element of pain perception is the adaptation of the patient’s tissues and emotions to contractions and the lack of time to accept them when labor progresses so dynamically. Patients in the Dinoprostone group developed contractile function later, and oxytocin was administered more often after 24 h, according to the schedule. Those patients had time to adapt to labor signs and symptoms (including pain). At the same time, we assume that this time was sufficient for cervical tissue changes during the cervical ripening process. Slow tissue adaptation during cervical maturation and slow-progressing contractions resulted in a longer time to delivery, but this time was still within the scheduled 48 hours. These patients required the addition of oxytocin, but at the same time, in most cases, the patients requested opioids less often, so we assume that delivery was less painful. We do not support the pursuit of dynamic labor following induction. Trying to outdo each other with ever-shorter times from the start of labor induction to birth is unnecessary. Therefore, as clinicians, we find the regimen with Dinoprostone more harmonious and desirable.

## Figures and Tables

**Table 1:** Comparative analysis of two groups: Dinoprostone vs. Misoprostol (up to 37 weeks of gestation, 37 to 41 weeks of gestation, and after 41 weeks of gestation): parturition type.

	Dinoprostone (n = 212)	Misoprostol (n = 190)	All (n = 402)	*p*-Value ^1^
<37 weeks of gestation (n = 53/42)				0.3536
Vaginal delivery (VD)	34 (64.2%)	23 (54.8%)	57 (60.0%)	
Cesarean section (CS)	19 (35.8%)	19 (45.2%)	38 (40.0%)	
37 to 41 weeks of gestation (n = 86/76)				0.0394
Vaginal delivery (VD)	63 (73.3%)	44 (57.9%)	107 (66.0%)	
Cesarean section (CS)	23 (26.7%)	32 (42.1%)	55 (34.0%)	
>41 weeks of gestation (n = 73/72)				0.8134
Vaginal delivery (VD)	46 (63.0%)	44 (61.1%)	90 (62.1%)	
Cesarean section (CS)	27 (37.0%)	28 (38.9%)	55 (37.9%)	

^1^ Chi-square.

**Table 2:** Comparative analysis of two groups: Dinoprostone vs. Misoprostol (up to 37 weeks of gestation, 37 to 41 weeks of gestation, and after 41 weeks of gestation): oxytocin augmentation, analgesia necessity, and occurrence of hyperstimulation.

	Dinoprostone (n = 212)	Misoprostol (n = 190)	All (n = 402)	*p*-Value ^1^
OXT augmentation				
<37 weeks (n = 53/42)	43 (81.1%)	15 (35.7%)	58 (61.1%)	<0.0001
37 to 41 weeks (n = 86/76)	57 (66.3%)	15 (19.7%)	72 (44.4%)	<0.0001
>41 weeks (n = 73/72)	43 (58.9%)	8 (11.1%)	51 (35.2%)	<0.0001
Intrapartum analgesia				
<37 weeks (n = 53/42)	40 (75.5%)	39 (92.9%)	79 (83.2%)	0.0245
37 to 41 weeks(n = 86/76)	61 (70.9%)	70 (92.1%)	131 (80.9%)	0.0006
>41 weeks (n = 73/72)	50 (68.5%)	64 (88.9%)	114 (78.6%)	0.0027
Hyperstimulation				
<37 weeks (n = 53/42)	0 (0.0%)	2 (4.8%)	2 (2.1%)	0.1084
37 to 41 weeks (n = 86/76)	2 (2.3%)	6 (7.9%)	8 (4.9%)	0.1025
>41 weeks (n = 73/72)	1 (1.4%)	4 (5.6%)	5 (3.4%)	0.1672

^1^ Chi-square.

**Table 3:** Comparative analysis of two groups, Dinoprostone vs. Misoprostol, in terms of preterm delivery (<37 weeks of gestation): time to delivery and parturition type.

	Dinoprostone (n = 53)	Misoprostol (n = 42)	*p*-Value ^1^
Time to delivery (VD and CS)			<0.0001
24 h	9 (17.0%)	27 (64.3%)	
24–48 h	32 (60.4%)	6 (14.3%)	
>48 h	12 (22.6%)	9 (21.4%)	
Parturition type			0.3536
Vaginal delivery (VD)	34 (64.2%)	23 (54.8%)	
Cesarean section (CS)	19 (35.8%)	19 (45.2%)	

^1^ Chi-square.

**Table 4:** Comparative analysis of two groups, Dinoprostone vs. Misoprostol, in terms of preterm delivery (<37 weeks of gestation): time to vaginal delivery.

	Dinoprostone (n = 34)	Misoprostol (n = 23)	*p*-Value ^1^
Time to VD			0.0004
24 h	5 (14.7%)	15 (65.2%)	
24–48 h	22 (64.7%)	5 (21.7%)	
>48 h	7 (20.6%)	3 (13.0%)	

^1^ Chi-square.

**Table 5:** Comparative analysis of two groups, Dinoprostone vs. Misoprostol, in terms of preterm delivery (<37 weeks of gestation): time to cesarean delivery.

	Dinoprostone (n = 19)	Misoprostol (n = 19)	*p*-Value ^1^
Time to CS			0.0033
24 h	4 (21.1%)	12 (63.2%)	
24–48 h	10 (52.6%)	1 (5.3%)	
>48 h	5 (26.3%)	6 (31.6%)	

^1^ Chi-square.

**Table 6:** Comparative analysis of two groups, Dinoprostone vs. Misoprostol, in terms of term delivery (37 to 41 weeks of gestation): time to delivery and parturition type.

	Dinoprostone (n = 86)	Misoprostol (n = 76)	*p*-Value ^1^
Time to delivery (VD and CS)			<0.0001
24 h	29 (33.7%)	61 (80.3%)	
24–48 h	46 (53.5%)	7 (9.2%)	
>48 h	11 (12.8%)	8 (10.5%)	
Parturition type			0.0394
Vaginal delivery (VD)	63 (73.3%)	44 (57.9%)	
Cesarean section (CS)	23 (26.7%)	32 (42.1%)	

^1^ Chi-square.

**Table 7:** Comparative analysis of two groups, Dinoprostone vs. Misoprostol, in terms of preterm delivery (37 to 41 weeks of gestation): time to vaginal delivery.

	Dinoprostone (n = 63)	Misoprostol (n = 44)	*p*-Value ^1^
Time to VD			<0.0001
24 h	20 (31.7%)	41 (93.2%)	
24–48 h	37 (58.7%)	1 (2.3%)	
>48 h	6 (9.5%)	2 (4.5%)	

^1^ Chi-square.

**Table 8:** Comparative analysis of two groups, Dinoprostone vs. Misoprostol, in terms of term delivery (37 to 41 weeks of gestation): time to cesarean delivery.

	Dinoprostone (n = 23)	Misoprostol (n = 32)	*p*-Value ^1^
Time to CS			0.1752
24 h	9 (39.1%)	20 (62.5%)	
24–48 h	9 (39.1%)	6 (18.8%)	
>48 h	5 (21.7%)	6 (18.8%)	

^1^ Chi-square.

**Table 9:** Comparative analysis of two groups, Dinoprostone vs. Misoprostol, in terms of term delivery (>41 weeks of gestation): time to delivery and parturition type.

	Dinoprostone (n = 73)	Misoprostol (n = 72)	*p*-Value ^1^
Time to delivery (VD and CS)			<0.0001
24 h	30 (41.1%)	64 (88.9%)	
24–48 h	33 (45.2%)	5 (6.9%)	
>48 h	10 (13.7%)	3 (4.2%)	
Parturition type			0.8134
Vaginal delivery (VD)	46 (63.0%)	44 (61.1%)	
Cesarean delivery (CS)	27 (37.0%)	28 (38.9%)	

^1^ Chi-square.

**Table 10:** Comparative analysis of two groups, Dinoprostone vs. Misoprostol, in terms of term delivery (>41 weeks of gestation): time to vaginal delivery.

	Dinoprostone (n = 46)	Misoprostol (n = 44)	*p*-Value ^1^
Time to VD			<0.0001
24 h	19 (41.3%)	43 (97.7%)	
24–48 h	26 (56.5%)	1 (2.3%)	
>48 h	1 (2.2%)	0 (0.0%)	

^1^ Chi-square.

**Table 11:** Comparative analysis of two groups, Dinoprostone vs. Misoprostol, in terms of term delivery (>41 weeks of gestation): time to cesarean delivery.

	Dinoprostone (n = 27)	Misoprostol (n = 28)	*p*-Value ^1^
Time to CS			0.0313
24 h	11 (40.7%)	21 (75.0%)	
24–48 h	7 (25.9%)	4 (14.3%)	
>48 h	9 (33.3%)	3 (10.7%)	

^1^ Chi-square.

## Data Availability

Data sharing is not applicable to this article due to the risk of the possibility of deanonymization.

## References

[1] Tsakiridis I., Mamopoulos A., Athanasiadis A., Dagklis T. (2020). Induction of Labor: An Overview of Guidelines. Obstet. Gynecol. Surv..

[2] Declercq E., Belanoff C., Iverson R. (2020). Maternal perceptions of the experience of attempted labor induction and medically elective inductions: Analysis of survey results from listening to mothers in California. BMC Pregnancy Childbirth.

[3] Grobman W.A., Rice M.M., Reddy U.M., Tita A.T.N., Silver R.M., Mallett G., Hill K., Thom E.A., El-Sayed Y.Y., Perez-Delboy A. (2018). Eunice Kennedy Shriver National Institute of Child Health and Human Development Maternal–Fetal Medicine Units Network. Labor Induction versus Expectant Management in Low-Risk Nulliparous Women. N. Engl. J. Med..

[4] Chen W., Xue J., Peprah M.K., Wen S.W., Walker M., Gao Y., Tang Y. (2016). A systematic review and network meta-analysis comparing the use of Foley catheters, Misoprostol, and Dinoprostone for cervical ripening in the induction of labor. BJOG.

[5] Liu Y.R., Pu C.X., Wang X.Y., Wang X.-Y. (2019). Double-balloon catheter versus dinoprostone insert for labor induction: A meta-analysis. Arch. Gynecol. Obstet..

[6] Hapangama D., Neilson J.P. (2009). Mifepristone for induction of labor. Cochrane Database Syst. Rev..

[7] Jones M.N., Palmer K.R., Pathirana M.M., Cecatti J.G., Filho O.B.M., Marions L., Edlund M., Prager M., Pennell C., Dickinson J.E. (2022). Balloon catheters versus vaginal prostaglandins for labor induction (CPI Collaborative): An individual participant data meta-analysis of randomised controlled trials. Lancet.

[8] Bakker R., Pierce S., Myers D. (2017). The role of prostaglandins E1 and E2, Dinoprostone, and Misoprostol in cervical ripening and the induction of labor: A mechanistic approach. Arch. Gynecol. Obstet..

[9] Narumiya S., Sugimoto Y., Ushikubi F. (1999). Prostanoid receptors: Structures, properties, and functions. Physiol. Rev..

[10] Austin S.C., Sanchez-Ramos L., Adair C.D. (2010). Labor induction with intravaginal Misoprostol compared with the dinoprostone vaginal insert: A systematic review and metaanalysis. Am. J. Obstet. Gynecol..

[11] Young D.C., Delaney T., Armson B.A., Fanning C. (2020). Oral misoprostol, low dose vaginal misoprostol, and vaginal Dinoprostone for labor induction: Randomized controlled trial. PLoS ONE.

[12] Pierce S., Bakker R., Myers D.A., Edwards R.K. (2018). Clinical Insights for Cervical Ripening and Labor Induction Using Prostaglandins. AJP Rep..

[13] Thomas J., Fairclough A., Kavanagh J., Kelly A.J. (2014). Vaginal prostaglandin (PGE2 and PGF2a) for induction of labor at term. Cochrane Database Syst. Rev..

[14] Papanikolaou E.G., Plachouras N., Drougia A., Andronikou S., Vlachou C., Stefos T., Paraskevaidis E., Zikopoulos K. (2004). Comparison of Misoprostol and Dinoprostone for elective induction of labor in nulliparous women at full term: A randomized prospective study. Reprod. Biol. Endocrinol..

[15] Chatsis V., Frey N. (2018). Misoprostol for Cervical Ripening and Induction of Labour: A Review of Clinical Effectiveness, Cost-Effectiveness and Guidelines.

[16] Swift E.M., Gunnarsdottir J., Zoega H., Bjarnadottir R.I., Steingrimsdottir T., Einarsdottir K. (2022). Trends in labor induction indications: A 20-year population-based study. Acta Obstet. Gynecol. Scand..

[17] Hodnett E.D., Lowe N.K., Hannah M.E., Willan A.R., Stevens B., Weston J.A., Ohlsson A., Gafni A., Muir H.A., Myhr T.L. (2002). Nursing Supportive Care in Labor Trial Group. Effectiveness of nurses as providers of birth labor support in North American hospitals: A randomized controlled trial. JAMA.

[18] Sydsjö G., Blomberg M., Palmquist S., Angerbjörn L., Bladh M., Josefsson A. (2015). Effects of continuous midwifery labor support for women with severe fear of childbirth. BMC Pregnancy Childbirth.

[19] Maputle M.S. (2018). Support provided by midwives to women during labor in a public hospital, Limpopo Province, South Africa: A participant observation study. BMC Pregnancy Childbirth.

[20] Anh N.D., Duc T.A., Ha N.T., Giang D.T., Dat D.T., Thuong P.H., Toan N.K., Duc N.T., Duc N.M. (2022). Dinoprostone Vaginal Insert for Induction of Labor in Women with Low-Risk Pregnancies: A Prospective Study. Med. Arch..

[21] Weeks A.D., Lightly K., Mol B.W., Frohlich J., Pontefract S., Williams M.J. (2022). Royal College of Obstetricians and Gynaecologists. Evaluating Misoprostol and mechanical methods for induction of labor: Scientific Impact Paper No. 68 April 2022. BJOG.

[22] Alfirevic Z., Keeney E., Dowswell T., Welton N.J., Dias S., Jones L.V., Navaratnam K., Caldwell D.M. (2015). Labour induction with prostaglandins: A systematic review and network meta-analysis. BMJ.

[23] Sugimoto Y., Narumiya S. (2007). Prostaglandin E receptors. J. Biol. Chem..

[24] Nicoll A. (2001). The Physiology of Cervical Ripening and the Induction of Labour: A Potential Role for the Nitric Oxide Donor Isosorbide Mononitrate. Master’s Thesis.

[25] Redling K., Schaedelin S., Huhn E.A., Hoesli I. (2019). Efficacy and safety of Misoprostol vaginal insert vs. oral Misoprostol for induction of labor. J. Perinat. Med..

[26] Hofmeyr G.J., Gülmezoglu A.M., Pileggi C. (2010). Vaginal misoprostol for cervical ripening and induction of labor. Cochrane Database Syst. Rev..

[27] Gattás D.S.M.B., de Amorim M.M.R., Feitosa F.E.L., da Silva-Junior J.R., Ribeiro L.C.G., Souza G.F.A., Souza A.S.R. (2020). Misoprostol administered sublingually at a dose of 12.5 μg versus vaginally at a dose of 25 μg for the induction of full-term labor: A randomized controlled trial. Reprod. Health.

[28] Czech I., Fuchs P., Fuchs A., Lorek M., Tobolska-Lorek D., Drosdzol-Cop A., Sikora J. (2018). Pharmacological and Nonpharmacological Methods of Labour Pain Relief-Establishment of Effectiveness and Comparison. Int. J. Environ. Res. Public Health.

[29] Santana L.S., Gallo R.B., Ferreira C.H., Duarte G., Quintana S.M., Marcolin A.C. (2016). Transcutaneous electrical nerve stimulation (TENS) reduces pain and postpones the need for pharmacological analgesia during labor: A randomised trial. J. Physiother..

[30] Levett K.M., Smith C.A., Dahlen H.G., Bensoussan A. (2014). Acupuncture and acupressure for pain management in labor and birth: A critical narrative review of current systematic review evidence. Complement Ther. Med..

[31] Beyable A.A., Bayable S.D., Ashebir Y.G. (2022). Pharmacologic and non-pharmacologic labor pain management techniques in a resource-limited setting: A systematic review. Ann. Med. Surg. (Lond).

[32] Zuarez-Easton S., Erez O., Zafran N., Carmeli J., Garmi G., Salim R. (2023). Pharmacologic and nonpharmacologic options for pain relief during labor: An expert review. Am. J. Obstet. Gynecol..

[33] Eberle R.L., Norris M.C. (1996). Labour analgesia: A risk-benefit analysis. Drug. Saf..

[34] Aziato L., Kyei A.A., Deku G. (2017). Experiences of midwives on pharmacological and nonpharmacological labor pain management in Ghana. Reprod. Health.

[35] Borders N., Wendland C., Haozous E., Leeman L., Rogers R. (2013). Midwives’ verbal support of nulliparous women in second-stage labor. J. Obstet. Gynecol. Neonatal. Nurs..

[36] Nilsson L., Thorsell T., Hertfelt Wahn E., Ekström A. (2013). Factors influencing positive birth experiences of first-time mothers. Nurs. Res. Pract..

[37] Hildingsson I., Westlund K., Wiklund I. (2013). Burnout in Swedish midwives. Sex. Reprod. Healthc..

[38] Tournaire M., Theau-Yonneau A. (2007). Complementary and alternative approaches to pain relief during labor. Evid. Based Complement. Altern. Med..

[39] Lei X., Yu Y., Li M., Fang P., Gan S., Yao Y., Zhou Y., Kang X. (2022). The efficacy and safety of Remifentanil patient-controlled versus epidural analgesia in labor: A meta-analysis and systematic review. PLoS ONE.

[40] Blajic I., Zagar T., Semrl N., Umek N., Lucovnik M., Stopar Pintaric T. (2021). Analgesic efficacy of Remifentanil patient-controlled analgesia versus combined spinal-epidural technique in multiparous women during labor. Ginekol. Pol..

[41] Egarter C.H., Husslein P.W., Rayburn W.F. (1990). Uterine hyperstimulation after low-dose prostaglandin E2 therapy: Tocolytic treatment in 181 cases. Am. J. Obstet. Gynecol..

[42] Bebbington M., Pevzner L., Schmuel E., Bernstein P., Dayal A., Barnhard J., Chazotte C., Merkatz I. (2003). Uterine tachysystole and hyperstimulation during induction of labor. Am. J. Obstet. Gynecol..

[43] Schmidt M., Neophytou M., Hars O., Freudenberg J., Kühnert M. (2019). Clinical experience with Misoprostol vaginal insert for induction of labor: A prospective clinical observational study. Arch. Gynecol. Obstet..

[44] Rouzi A.A., Alsibiani S., Mansouri N., Alsinani N., Darhouse K. (2014). Randomized clinical trial between hourly titrated oral Misoprostol and vaginal Dinoprostone for induction of labor. Am. J. Obstet. Gynecol..

[45] Rugarn O., Tipping D., Powers B., Wing D.A. (2017). Induction of labor with retrievable prostaglandin vaginal inserts: Outcomes following retrieval due to an intrapartum adverse event. BJOG.

[46] Mlodawski J., Mlodawska M., Armanska J., Swiercz G., Gluszek S. (2021). Misoprostol vs. dinoprostone vaginal insert in labor induction: Comparison of obstetrical outcome. Sci. Rep..

[47] Nicholson J., Kellar L., Henning G., Waheed A., Colon-Gonzalez M., Ural S. (2015). The association between the regular use of preventive labor induction and improved term birth outcomes: Findings of a systematic review and meta-analysis. BJOG Int. J. Obstet. Gynaecol..

[48] ACOG (2009). ACOG Practice Bulletin No. 107: Induction of labor. Obstet. Gynecol..

[49] Zahran K.M., Shahin A.Y., Abdellah M.S., Elsayh K.I. (2009). Sublingual versus vaginal Misoprostol for induction of labor at term: A randomized prospective placebo-controlled study. J. Obstet. Gynaecol. Res..

[50] Sharami S.H., Milani F., Faraji R., Bloukimoghadam K., Salamat F., Momenzadeh S., Ebrahimi H. (2014). Comparison of 25 µg sublingual and 50 µg intravaginal misoprostol for cervical ripening and labor: A randomized controlled equivalence trial. Arch. Iran. Med..

[51] Nassar A.H., Awwad J., Khalil A.M., Abu-Musa A., Mehio G., Usta I.M. (2007). A randomised comparison of patient satisfaction with vaginal and sublingual Misoprostol for induction of labour at term. BJOG.

[52] Singer A., Jordan J.A. (2009). The Functional Anatomy of the Cervix, the Cervical Epithelium and the Stroma. The Cervix.

[53] Garavito R.M., Dewitt D.L. (1999). The Cyclooxygenase Isoforms: Structural Insights into the Conversion of Arachidonic Acid to Prostaglandins. Biochim. Biophys. Acta.

[54] Roos N., Blesson C.S., Stephansson O., Masironi B., Vladic Stjernholm Y., Ekman-Ordeberg G., Sahlin L. (2014). The expression of prostaglandin receptors EP3 and EP4 in human cervix in post-term pregnancy differs between failed and successful labor induction. Acta Obstet. Gynecol. Scand..

[55] El Maradny E., Kanayama N., Halim A., Maehara K., Sumimoto K., Terao T. (1996). Biochemical changes in the cervical tissue of rabbit induced by interleukin-8, interleukin-1beta, dehydroepiandrosterone sulphate and prostaglandin E2: A comparative study. Hum. Reprod..

[56] Erkinheimo T.-L., Saukkonen K., Narko K., Jalkanen J., Ylikorkala O., Ristimäki A. (2000). Expression of Cyclooxygenase-2 and Prostanoid Receptors by Human Myometrium. J. Clin. Endocrinol. Metab..

[57] Oliveira T.A., Melo E.M., Aquino M.M., Mariani Neto C. (2011). Eficácia de dinoprostone e misoprostol para indução do trabalho de parto em nulíparas [Efficacy of dinoprostone and misoprostol for labor induction in nulliparous women]. Rev. Bras. Ginecol. Obstet..

[58] Aghideh F.K., Mullin P.M., Ingles S., Ouzounian J.G., Opper N., Wilson M.L., Miller D.A., Lee R.H. (2014). A comparison of obstetrical outcomes with labor induction agents used at term. J. Matern. Fetal. Neonatal. Med..

[59] Wing D.A., Brown R., Plante L.A., Miller H., Rugarn O., Powers B.L. (2013). Misoprostol vaginal insert and time to vaginal delivery: A randomized controlled trial. Obstet. Gynecol..

[60] Maggi C., Mazzoni G., Gerosa V., Fratelli N., Prefumo F., Sartori E., Lojacono A. (2019). Labor induction with Misoprostol vaginal insert compared with dinoprostone vaginal insert. Acta Obstet. Gynecol. Scand..

[61] Bomba-Opoń D., Drews K., Huras H., Laudański P., Paszkowski T., Wielgoś M. (2017). Polish Gynecological Society Recommendations for Labor Induction. Ginekol. Pol..

